# Supplementary material for: Upgraded molecular models of the human KCNQ1 potassium channel
Source: PLoS One. 2019 Sep 13;14(9):e0220415. doi: 10.1371/journal.pone.0220415 (PMC6743773; doi:10.1371/journal.pone.0220415)
Supplement: S1 Table — (DOCX) [file pone.0220415.s001.docx]

| **S1 Table: Molprobity statistic of KCNQ1 AO, RC and AC homology models** | | | |
| --- | --- | --- | --- |
|  | **KCNQ1 AO state** | **KCNQ1 RC state** | **KCNQ1 AC state** |
| Molprobity score | 1.38 (97^th^ percentile) | 1.71 (90^th^ percentile) | 1.12 (100^th^ percentile) |
| Clash score | 3.09 (98^th^ percentile) | 4.92 (94^th^ percentile) | 2.72 (98^th^ percentile) |
| Ramachandran statistic |  |  |  |
| Favored regions (%) | 95.9 | 92.8 | 97.7 |
| Allowed regions (%) | 4.1 | 6.2 | 1.9 |
| Disallowed regions (%) | 0.0 | 1.0 | 0.4 |
| Rotamer statistic |  |  |  |
| Favored rotamers (%) | 100.0 | 99.5 | 100.0 |
| Poor rotamers (%) | 0.0 | 0.0 | 0.0 |
| Cβ deviations | 0 | 0 | 0 |
| Bad bonds (%) | 0.00 | 0.00 | 0.00 |
| Bad angles (%) | 0.03 | 0.03 | 0.10 |
