# Supplementary material for: Upgraded molecular models of the human KCNQ1 potassium channel
Source: PLoS One. 2019 Sep 13;14(9):e0220415. doi: 10.1371/journal.pone.0220415 (PMC6743773; doi:10.1371/journal.pone.0220415)
Supplement: S2 Table — (DOCX) [file pone.0220415.s002.docx]

| **Table S2: Comparison of Rosetta predicted stability changes (ΔΔG) with expression levels, trafficking efficiencies and peak current densities of KCNQ1 VSD mutants** | | | | | | | | |
| --- | --- | --- | --- | --- | --- | --- | --- | --- |
| **KCNQ1 variant** | **ClinVar Class** | **Functional Class*** | **Total expression**  **(% WT)^#^** | **Cell surface expression**  **(% WT)** | **Trafficking efficiency**  **(% WT)** | **Peak current density**  **(% WT)** | **ΔΔG RC model**  **(REU)** | **ΔΔG AO model**  **(REU)** |
| V100I | neutral | VI | 101.1 ± 3.4 | 91.0 ± 4.4 | 112.4 ± 13.3 | 113.0 ± 14.0 | 0.7 ± 3.1 | 0.3 ± 2.2 |
| A102S | neutral | VI | 106.5 ± 4.9 | 95.8 ± 5.2 | 113.1 ± 16.8 | 282.0 ± 36.0 | -2.9 ± 1.7 | 0.2 ± 3.2 |
| T104I | VUS | IV | 89.0 ± 4.6 | 64.9 ± 5.1 | 90.7 ± 10.0 | 22.0 ± 4.0 | 2.2 ± 1.9 | 8.2 ± 2.7 |
| T104S | neutral | VI | 101.8 ± 5.0 | 93.2 ± 6.2 | 113.9 ± 11.8 | 82.0 ± 13.0 | 3.4 ± 1.3 | 5.5 ± 4.2 |
| H105L | LQTS | III | 97.6 ± 4.4 | 80.2 ± 7.5 | 101.6 ± 9.6 | 131.0 ± 17.0 | -5.5 ± 2.6 | 6.4 ± 2.1 |
| H105N | neutral | VI | 131.8 ± 6.2 | 105.6 ± 6.7 | 98.9 ± 4.9 | 72.0 ± 9.0 | -4.7 ± 1.8 | 0.0 ± 2.4 |
| H105Y | neutral | VI | 94.7 ± 2.6 | 78.4 ± 5.5 | 102.2 ± 7.5 | 151.0 ± 21.0 | 4.3 ± 4.3 | 6.8 ± 2.5 |
| V106I | neutral | VI | 121.4 ± 7.7 | 103.7 ± 4.2 | 106.7 ± 11.3 | 121.0 ± 18.0 | 1.2 ± 2.5 | 4.5 ± 1.4 |
| Q107H | VUS | II | 20.1 ± 2.5 | 5.6 ± 1.7 | 33.8 ± 8.5 | 36.0 ± 6.0 | 4.3 ± 3.7 | 12.6 ± 2.1 |
| R109L | VUS | III | 114.0 ± 2.8 | 91.9 ± 6.2 | 99.2 ± 4.5 | 223.0 ± 35.0 | 10.9 ± 2.6 | 10.3 ± 1.7 |
| V110I | LQTS | I | 114.1 ± 6.2 | 96.4 ± 5.5 | 104.8 ± 8.4 | 25.0 ± 5.0 | 3.2 ± 2.7 | 3.4 ± 2.2 |
| Y111C | LQTS | V | 17.6 ± 2.5 | 2.0 ± 1.1 | 13.5 ± 6.7 | -2.0 ± 3.0 | 19.5 ± 3.6 | 30.2 ± 2.3 |
| L114P | LQTS | V | 15.6 ± 2.1 | 2.0 ± 1.2 | 14.8 ± 7.7 | -1.0 ± 4.0 | 77.1 ± 4.0 | 70.5 ± 4.1 |
| E115G | LQTS | III | 18.5 ± 1.2 | 1.9 ± 1.0 | 12.6 ± 6.4 | -7.0 ± 3.0 | 27.6 ± 4.0 | 26.9 ± 4.6 |
| P117L | LQTS | V | 25.7 ± 2.5 | 5.5 ± 1.4 | 26.3 ± 5.9 | -5.0 ± 3.0 | 20.1 ± 5.9 | 24.2 ± 7.6 |
| T118S | neutral | III | 110.6 ± 1.5 | 100.7 ± 7.1 | 112.0 ± 4.7 | 76.0 ± 14.0 | 3.6 ± 1.9 | 0.5 ± 2.2 |
| C122Y | LQTS | I | 95.6 ± 10.8 | 71.7 ± 4.0 | 66.8 ± 4.0 | -4.0 ± 3.0 | -3.5 ± 2.8 | -11.9 ± 2.9 |
| V124I | neutral | VI | 112.1 ± 15.4 | 112.8 ± 3.5 | 79.8 ± 4.3 | 92.0 ± 12.0 | -1.5 ± 2.5 | -5.8 ± 1.8 |
| Y125D | VUS | V | 12.6 ± 1.4 | 2.1 ± 0.6 | 20.0 ± 5.4 | 1.0 ± 3.0 | 18.2 ± 1.7 | 27.6 ± 1.8 |
| H126L | VUS | V | 32.4 ± 3.3 | 3.7 ± 0.9 | 4.2 ± 2.6 | 4.0 ± 3.0 | 4.1 ± 3.2 | 11.5 ± 6.0 |
| F127L | LQTS | VI | 107.6 ± 4.4 | 110.7 ± 4.1 | 85.1 ± 13.1 | 80.0 ± 13.0 | 0.3 ± 2.3 | -6.4 ± 2.2 |
| A128T | neutral | VI | 101.8 ± 8.5 | 112.8 ± 4.7 | 95.3 ± 19.3 | 74.0 ± 11.0 | -2.2 ± 2.1 | 0.7 ± 2.3 |
| V129I | VUS | VI | 120.7 ± 8.2 | 132.0 ± 6.7 | 91.1 ± 13.6 | 83.0 ± 16.0 | -4.4 ± 2.8 | 1.6 ± 2.3 |
| L131P | VUS | IV | 41.9 ± 4.8 | 45.4 ± 4.0 | 102.4 ± 20.9 | 6.0 ± 3.0 | 61.5 ± 2.5 | 76.0 ± 3.0 |
| I132L | LQTS | III | 109.9 ± 6.2 | 104.4 ± 6.5 | 90.7 ± 2.9 | 112.0 ± 23.0 | -4.9 ± 2.2 | -0.2 ± 1.7 |
| V133I | LQTS | VI | 110.9 ± 6.7 | 95.0 ± 3.9 | 85.0 ± 1.1 | 67.0 ± 16.0 | 5.3 ± 2.5 | 0.6 ± 2.8 |
| L134P | LQTS | I | 60.0 ± 3.2 | 99.6 ± 11.4 | 177.7 ± 11.7 | 3.0 ± 2.0 | 63.7 ± 2.6 | 68.3 ± 4.1 |
| V135A | neutral | VI | 103.2 ± 4.7 | 92.3 ± 5.2 | 91.7 ± 1.3 | 78.0 ± 15.0 | 1.8 ± 3.0 | 5.4 ± 2.5 |
| V135I | neutral | VI | 99.1 ± 8.5 | 83.6 ± 7.6 | 92.7 ± 6.7 | 100.0 ± 11.0 | -7.0 ± 2.3 | -2.5 ± 2.3 |
| A149V | neutral | VI | 66.1 ± 2.7 | 73.4 ± 10.2 | 127.2 ± 3.5 | 107.0 ± 34.0 | 16.1 ± 3.7 | 26.5 ± 2.3 |
| A150T | VUS | I | 117.4 ± 7.5 | 156.1 ± 10.7 | 139.3 ± 1.1 | 58.0 ± 9.0 | 1.4 ± 2.1 | -4.4 ± 2.3 |
| A150V | neutral | I | 90.6 ± 5.5 | 96.8 ± 4.7 | 113.8 ± 9.7 | 52.0 ± 17.0 | 5.9 ± 1.6 | 3.4 ± 2.4 |
| E160K | LQTS | IV | 35.5 ± 3.0 | 15.8 ± 1.6 | 55.0 ± 3.5 | -4.0 ± 3.0 | 19.5 ± 4.2 | 16.3 ± 5.2 |
| T169M | VUS | I | 87.8 ± 4.5 | 86.5 ± 4.7 | 121.6 ± 5.2 | 65.0 ± 10.0 | -7.6 ± 1.9 | -4.4 ± 2.9 |
| R174C | LQTS | V | 14.0 ± 4.0 | 2.2 ± 1.2 | 17.2 ± 6.8 | 0.0 ± 2.0 | 40.0 ± 3.0 | 36.3 ± 3.5 |
| R174H | LQTS | V | 20.0 ± 2.7 | 4.2 ± 1.3 | 25.6 ± 6.2 | -1.0 ± 3.0 | 37.7 ± 2.0 | 37.1 ± 4.2 |
| R174L | LQTS | V | 17.5 ± 2.4 | 9.7 ± 1.8 | 68.0 ± 7.5 | 3.0 ± 3.0 | 27.0 ± 2.0 | 31.4 ± 4.1 |
| W176R | VUS | V | 10.8 ± 1.9 | 2.5 ± 1.7 | 24.1 ± 14.4 | 1.0 ± 2.0 | 14.7 ± 2.8 | 9.3 ± 3.6 |
| G179S | LQTS | V | 17.7 ± 3.2 | 4.1 ± 2.2 | 25.3 ± 12.8 | 10.0 ± 3.0 | -5.6 ± 1.9 | 2.6 ± 2.5 |
| G189A | VUS | V | 14.1 ± 0.8 | 2.4 ± 1.7 | 19.6 ± 13.3 | -5.0 ± 2.0 | 13.2 ± 1.6 | 21.6 ± 3.8 |
| R195P | VUS | V | 16.8 ± 2.8 | 2.0 ± 1.6 | 11.6 ± 8.6 | -1.0 ± 3.0 | 52.7 ± 1.1 | 56.8 ± 2.0 |
| K196T | VUS | IV | 61.6 ± 3.9 | 58.3 ± 2.8 | 116.9 ± 2.4 | 20.0 ± 5.0 | 1.0 ± 3.1 | 5.2 ± 3.4 |
| P197L | VUS | II | 68.1 ± 3.0 | 59.1 ± 3.7 | 107.3 ± 7.0 | 147.0 ± 23.0 | -11.6 ± 2.8 | -10.9 ± 2.6 |
| P197S | VUS | IV | 40.2 ± 4.4 | 21.2 ± 2.3 | 65.2 ± 0.3 | 17.0 ± 4.0 | 6.9 ± 1.4 | 10.7 ± 4.0 |
| V207M | LQTS | VI | 85.6 ± 4.4 | 70.8 ± 4.1 | 102.0 ± 2.7 | 88.0 ± 15.0 | 4.2 ± 4.3 | 4.9 ± 2.4 |
| K218E | VUS | I | 93.1 ± 5.3 | 101.8 ± 3.5 | 135.5 ± 7.8 | 46.0 ± 9.0 | 12.4 ± 1.7 | 8.4 ± 2.4 |
| I227L | VUS | I | 116.0 ± 7.5 | 142.9 ± 9.8 | 152.0 ± 6.1 | 22.0 ± 5.0 | -7.5 ± 2.2 | 4.4 ± 2.0 |
| Q234P | VUS | I | 116.4 ± 6.8 | 131.8 ± 1.5 | 140.1 ± 3.7 | 3.0 ± 4.0 | 82.1 ± 3.8 | 71.0 ± 5.0 |
| L236P | VUS | IV | 78.1 ± 4.7 | 47.0 ± 8.3 | 72.0 ± 7.3 | 3.0 ± 4.0 | 60.3 ± 4.8 | 65.8 ± 6.0 |
| L236R | VUS | IV | 56.1 ± 2.7 | 15.6 ± 3.5 | 33.5 ± 5.8 | 1.0 ± 4.0 | 13.9 ± 2.6 | 19.0 ± 3.2 |
| *Functional classification of KCNQ1 variants according to reference (1):  **Class I:** Normal or higher expression and trafficking levels but low peak current density. Dysfunctional channel.  **Class II:** Lower expression levels but normal channel function for channel variants that traffic to the membrane.  **Class III:** Normal or higher expression and trafficking levels and peak current density but altered channel V_1/2_ and/or deactivation rate.  **Class IV:** Defective in surface expression levels and electrophysiological channel properties.  **Class V:** Severe expression and/or trafficking defects. Current is so low that channel properties cannot be assessed.  **Class VI:** Have wildtype-like behavior. Normal or higher surface expression levels and channel properties.  ^#^ Experimental data are from reference (1). Values are reported as mean ± S.E.M. | | | | | | | | |
