# Supplementary figures and images for: Upgraded molecular models of the human KCNQ1 potassium channel
Source: PLoS One. 2019 Sep 13;14(9):e0220415. doi: 10.1371/journal.pone.0220415 (PMC6743773; doi:10.1371/journal.pone.0220415)

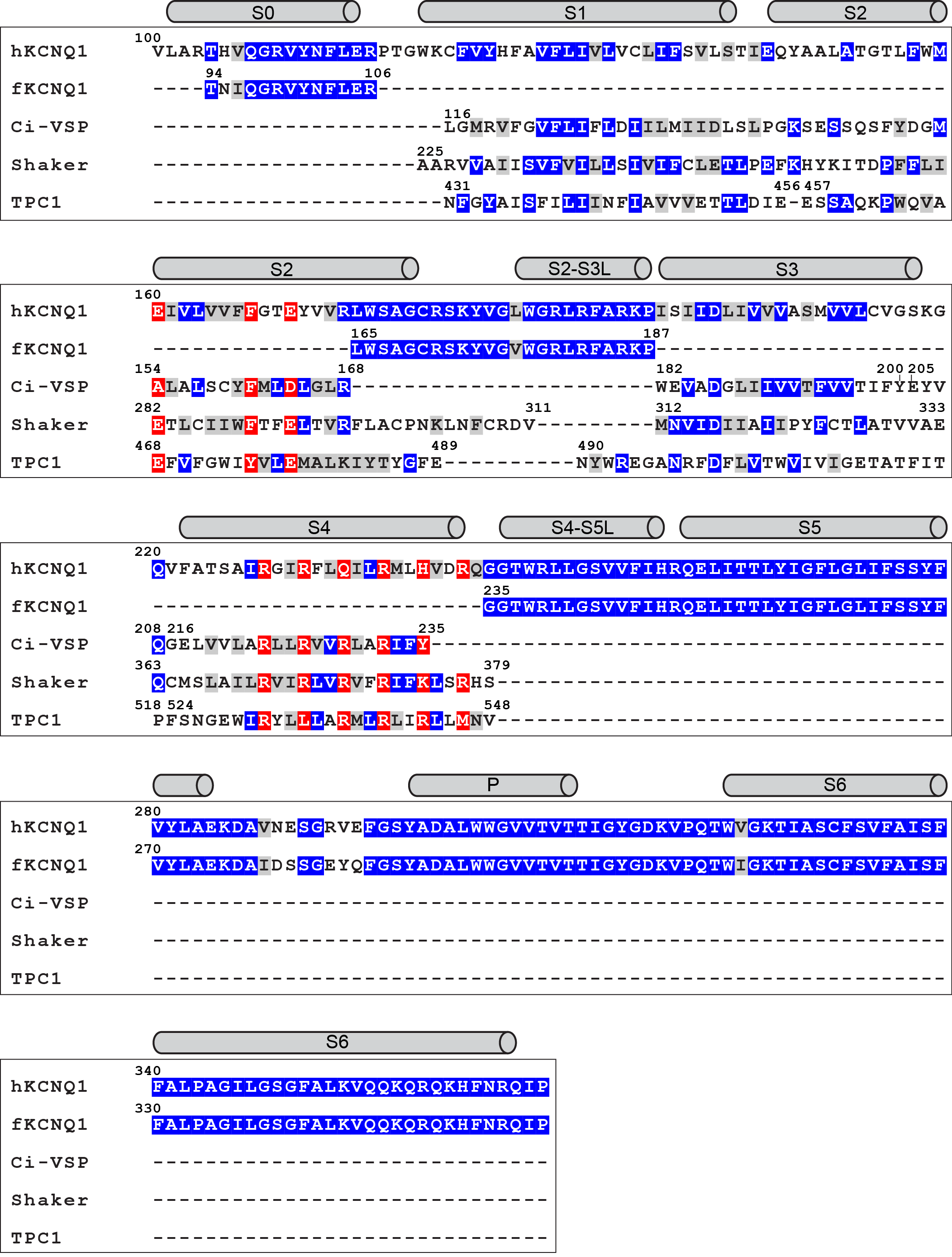

Supplement: S1 Fig — C. intestinalis voltage-sensing phosphatase (Ci-VSP) (PDB 4G7Y), VSD2 in A. thaliana two pore calcium channel protein 1 (TPC1) (PDB 5DQQ), and the resting VSD conformation C3 in a model of the Shaker K channel were used as structural templates for helix S1, S2, S3 and S4 of the VSD. The cryo-EM structure of X. leavis KCNQ1 (fKCNQ1) (PDB 5VMS) was used as template for helix S0, the S2-S3 linker and the pore domain. The alignment was created with MAMMOTH [118] and ClustalW [106] and manually adjusted to ensure that functionally conserved residues in S2, S3 and S4 are correctly aligned. Identical and similar residues are colored blue and gray, respectively. Residues at structurally conserved positions are highlighted in red. Predicted secondary structure regions are indicated above the sequence alignment as gray cylinders. (TIFF) [file pone.0220415.s003.tiff]

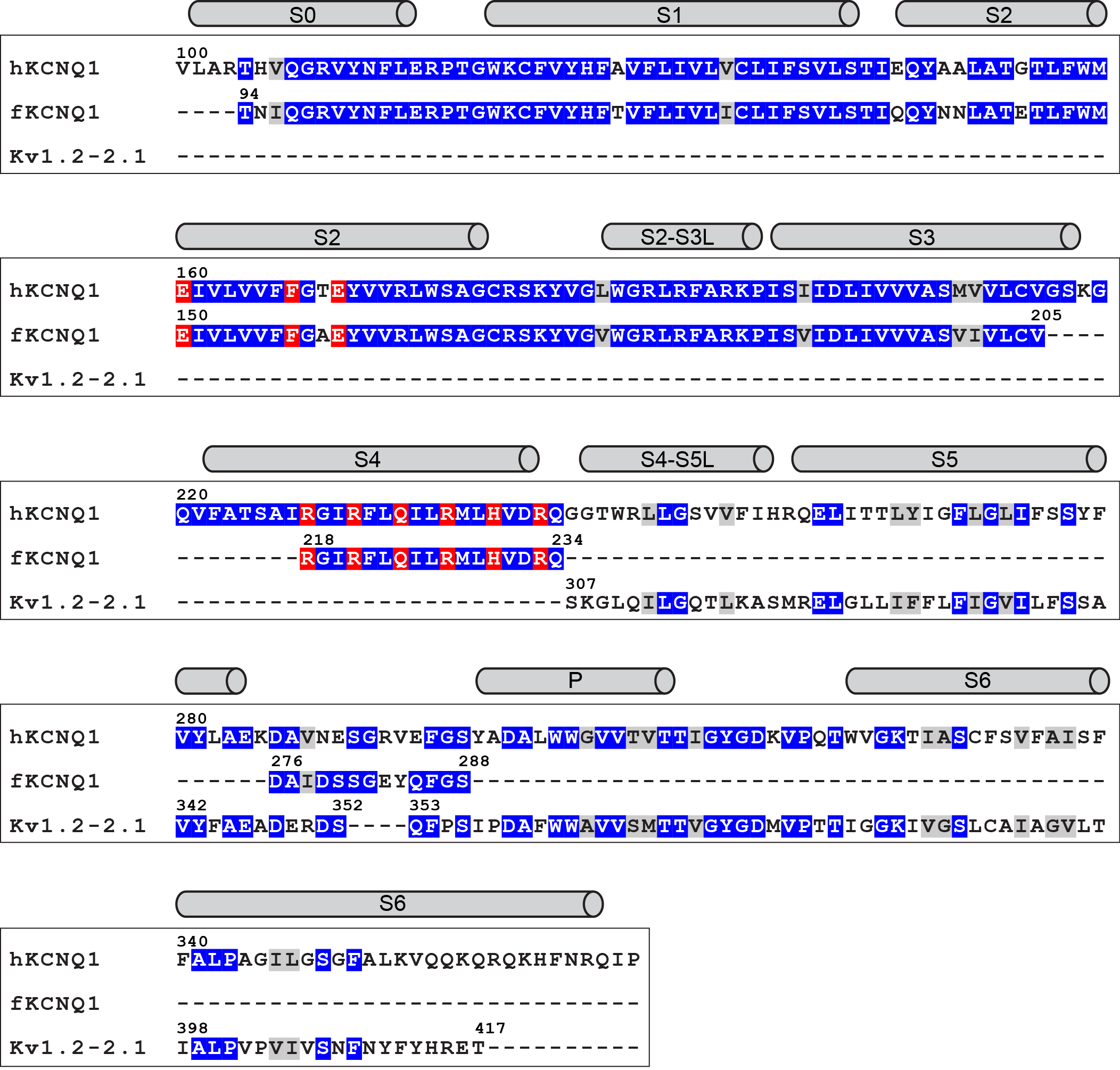

Supplement: S2 Fig — The cryo-EM structure of X. leavis KCNQ1 (fKCNQ1) (PDB 5VMS) and the X-ray structure of the rat chimeric Kv1.2–2.1 channel (PDB 2R9R) were used as structural templates. The alignment was created with MAMMOTH [118] and ClustalW [106] and manually adjusted to ensure functionally conserved residues in S2, S3 and S4 are correctly aligned. Identical and similar residues are colored blue and gray, respectively. Residues at structurally conserved positions are highlighted in red. Predicted secondary structure regions are indicated above the sequence alignment as gray cylinders. (TIFF) [file pone.0220415.s004.tiff]

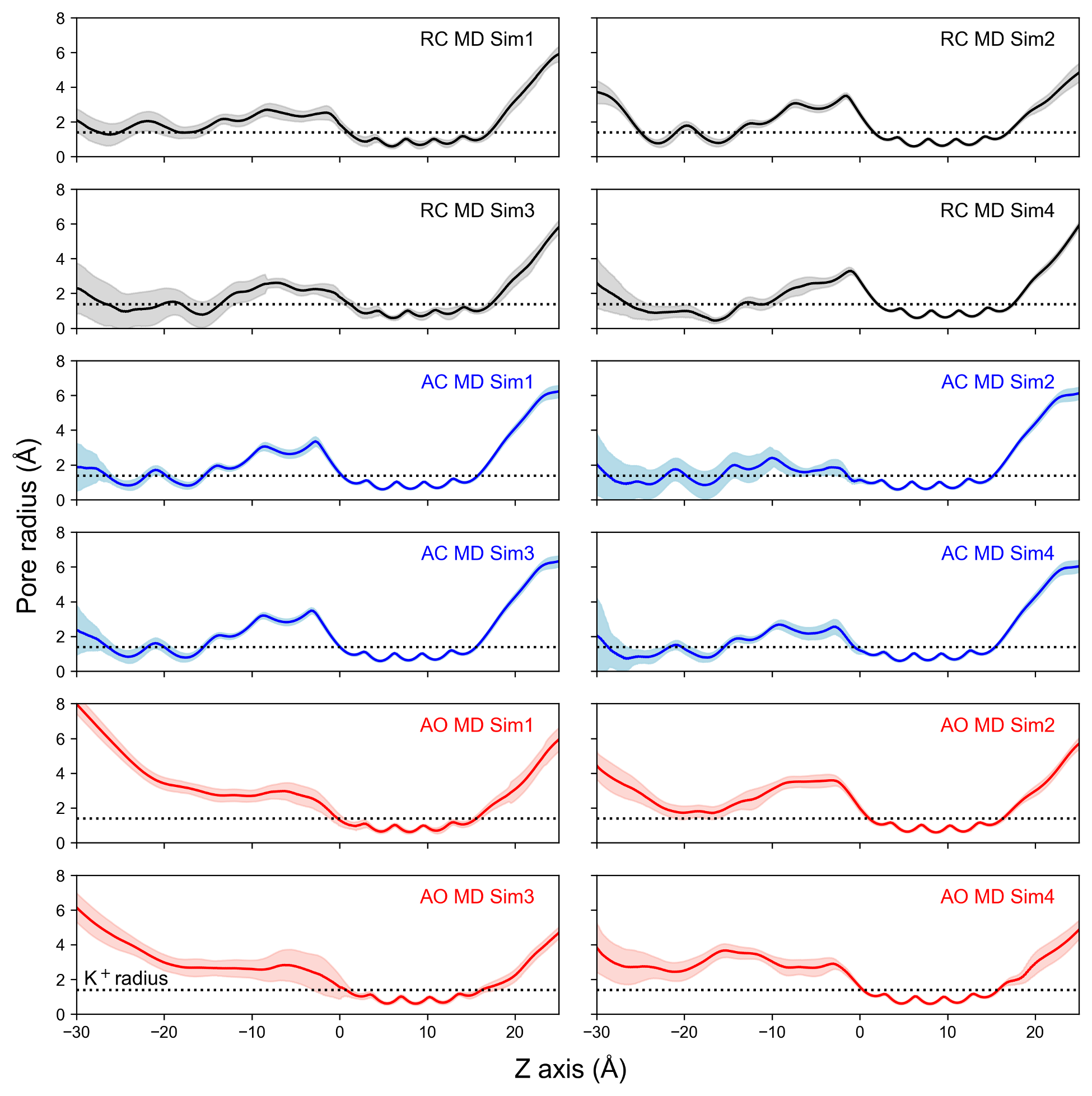

Supplement: S3 Fig — Four MD simulations of the RC, AC and AO state, respectively, were conducted in this study. The average pore radius was calculated for the configurations after the first 100 ns of production MD and is shown as solid line. Shaded areas correspond to one standard deviation. The approximate radius of a K+ ion is indicated as dashed line. The region between 3Å – 14Å corresponds to the channel selectivity filter. (TIFF) [file pone.0220415.s005.tiff]

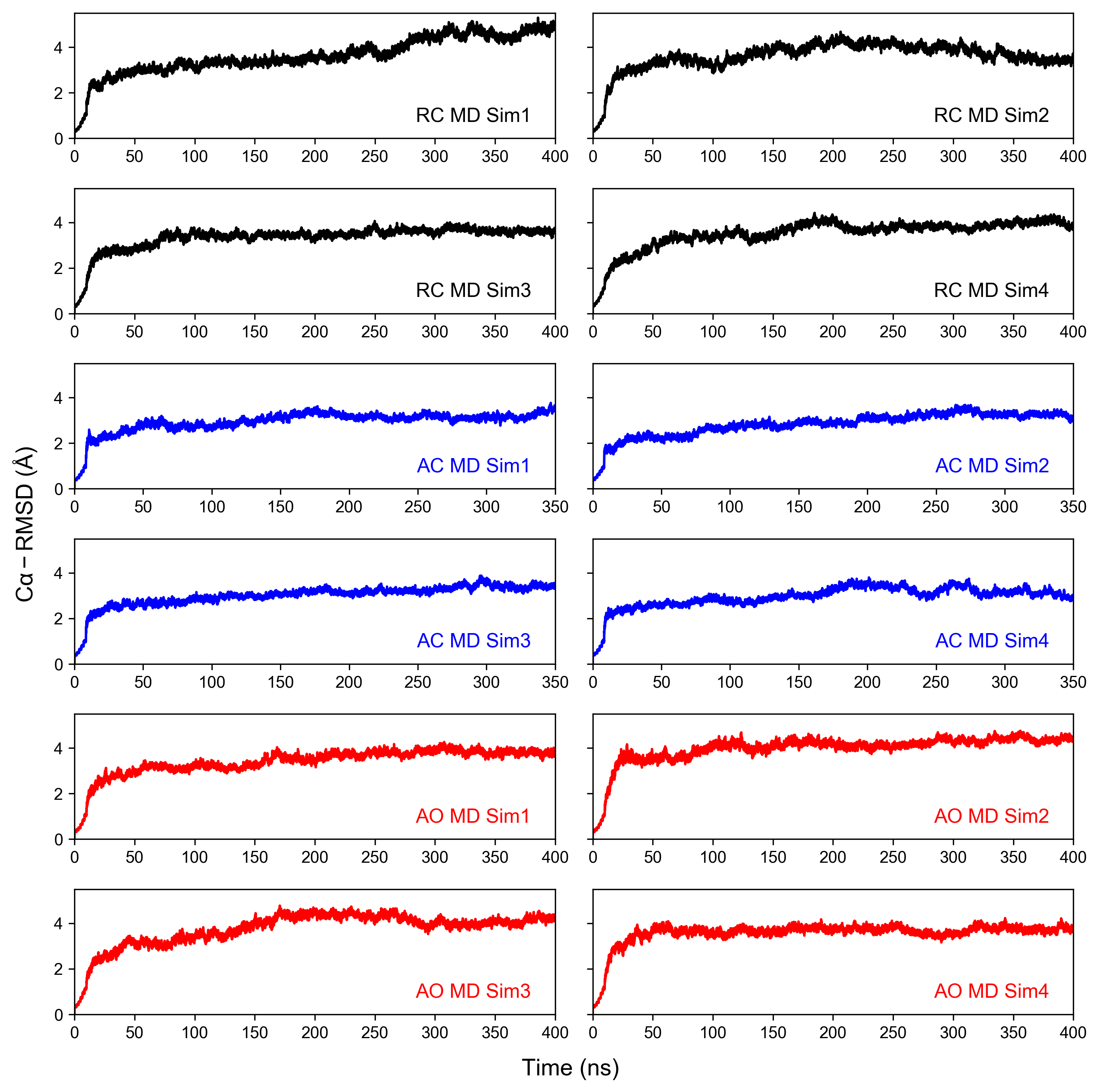

Supplement: S4 Fig — Four MD simulations of the RC, AC and AO state, respectively, were conducted in this study. Each simulation started from a different model from the final ensemble of 20–30 Rosetta homology models and was conducted for 400 ns (RC and AO) or 350 ns (AC) as described in Methods. The RMSD is displayed for the production period of MD and was calculated relative to the conformation after minimization and system heating. (TIFF) [file pone.0220415.s006.tiff]

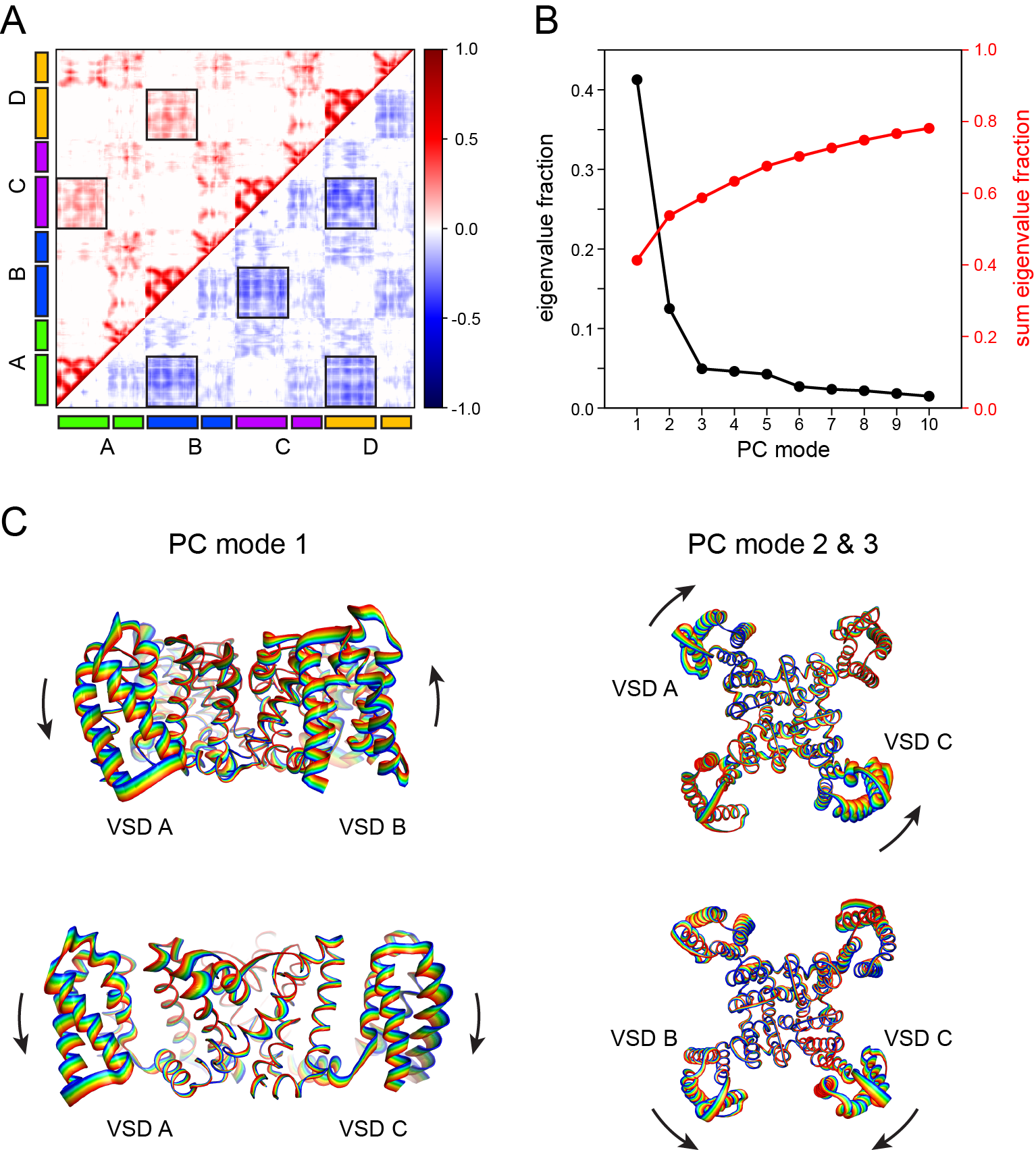

Supplement: S5 Fig — (A) Average dynamic cross-correlation matrix of the KCNQ1 tetramer calculated from MD trajectories of the AO models. Positive residue correlations are plotted in the upper triangular matrix whereas negative correlations are mapped on the lower triangle. Regions in the cross-correlation matrix corresponding to correlations between VSDs are framed by black boxes. The approximate regions of the four channel subunits (labeled A–D) are indicated on the x- and y-axis. (B) Scree plot of the first ten principal components obtained by PCA of the KCNQ1 MD simulations. (C) Pseudo-trajectories along the first three PC modes. PC mode 1 corresponds to a VSD movement along the membrane normal with two VSDs on the same side of the channel tetramer moving anti-parallel and VSDs on opposite sides moving in a parallel fashion. PC modes 2 and 3 represent a swing movement of the VSDs within the membrane plane. (TIFF) [file pone.0220415.s007.tiff]

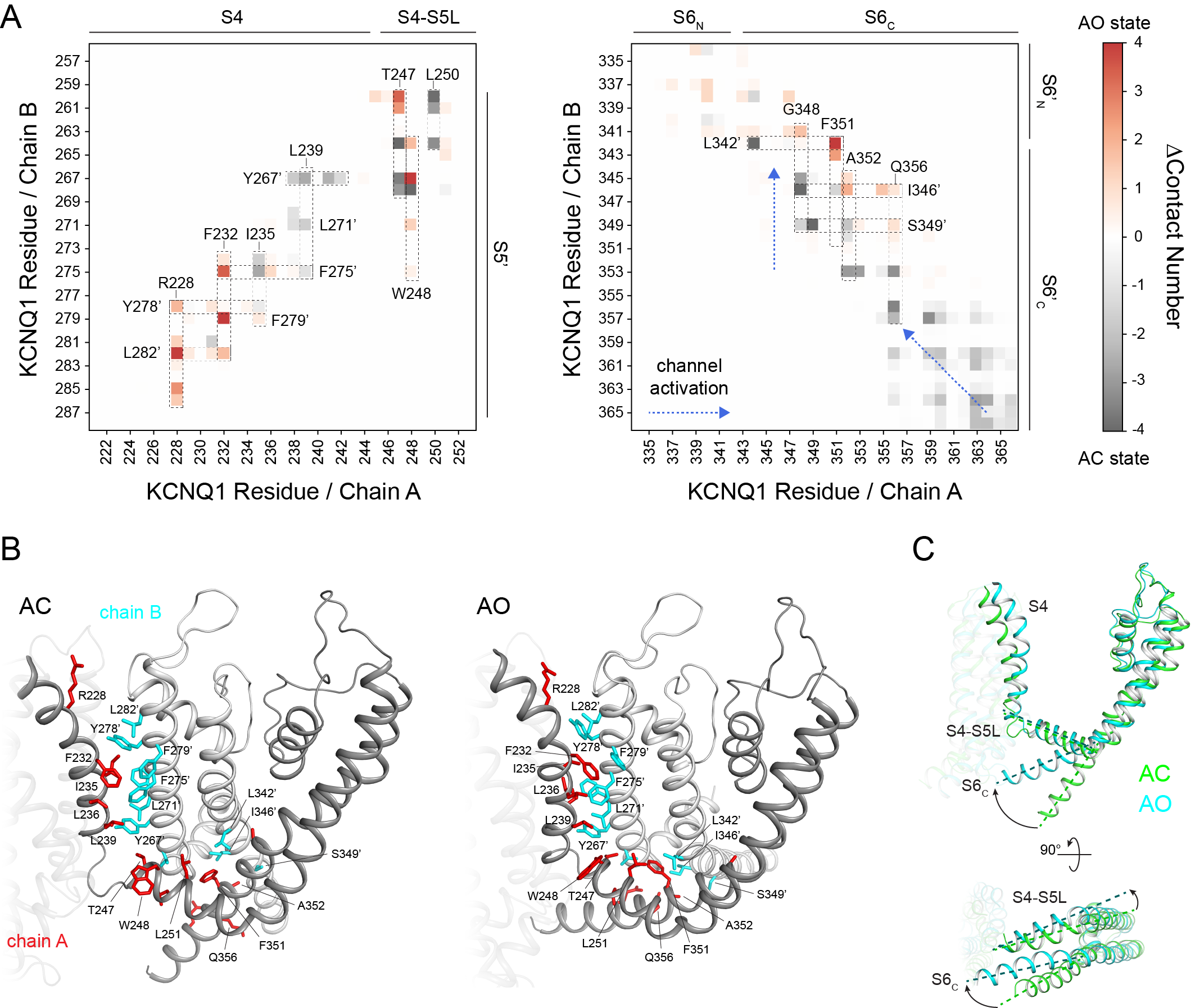

Supplement: S6 Fig — (A) Change in average contact number between residues in two neighboring KCNQ1 chains A and B. The contact number was defined as number of heteroatom pairs within a 4 Å distance and averaged over the last 250 ns of MD and all four pairs of neighboring subunits in the KCNQ1 tetramer. The section of the contact matrix corresponding to the interface of S4 and S4-S5L with S5’ (left) and of two neighboring helices S6 and S6’ (right) is shown. A gray color means this contact is observed in the AC state whereas a red color indicates a contact in the AO state. Changes in specific residue contacts occurring while the channel transitions from the AC to the AO state are framed and labeled by their corresponding amino acid residue. The direction of the structural changes with channel opening is indicated by a blue arrow. (B) Cartoon representation of the inter-subunit interface in the AC (left) and AO (right) model, respectively. For clarity, only helices S4 to S6 are shown. Residues which are part of the inter-subunit interface and fall within regions of the contact matrix in (A) are depicted as sticks. Residues with drastic changes in their contact pattern as identified in (A) are labeled. (C) Movement of helix S4-S5L and S6C during transition from the AC to AO state. Only one KCNQ1 subunit is displayed for clarity with the superimposition optimized for the pore domain. Helix axes are marked by dashed lines and the direction of their movement is indicated by arrows. The S4-S5 linker is in a similar upward position in the AC and AO state (upper cartoon), but moves sideward within the membrane plane (lower cartoon) in the AO state allowing S6C to bend and move outward. (TIFF) [file pone.0220415.s008.tiff]

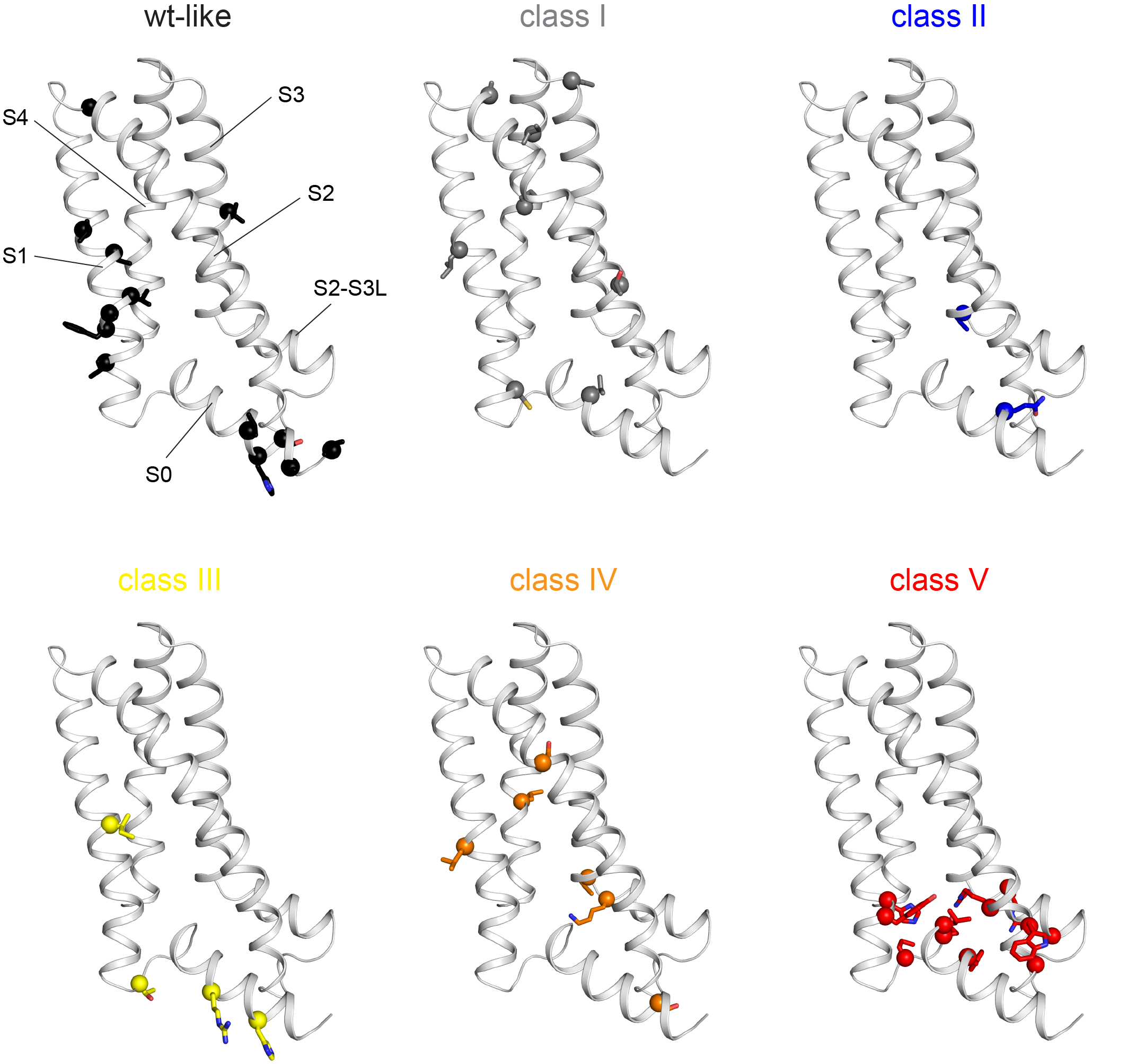

Supplement: S7 Fig — The VSD is represented as ribbon with helical segments labeled. The backbone position of mutation sites is indicated by a sphere and the native amino acid residue is shown in sticks. The assignment of variants to these six classes can be found in S2 Table and reference [19] and the class definition is given in the footnote of S2 Table. (TIFF) [file pone.0220415.s009.tiff]

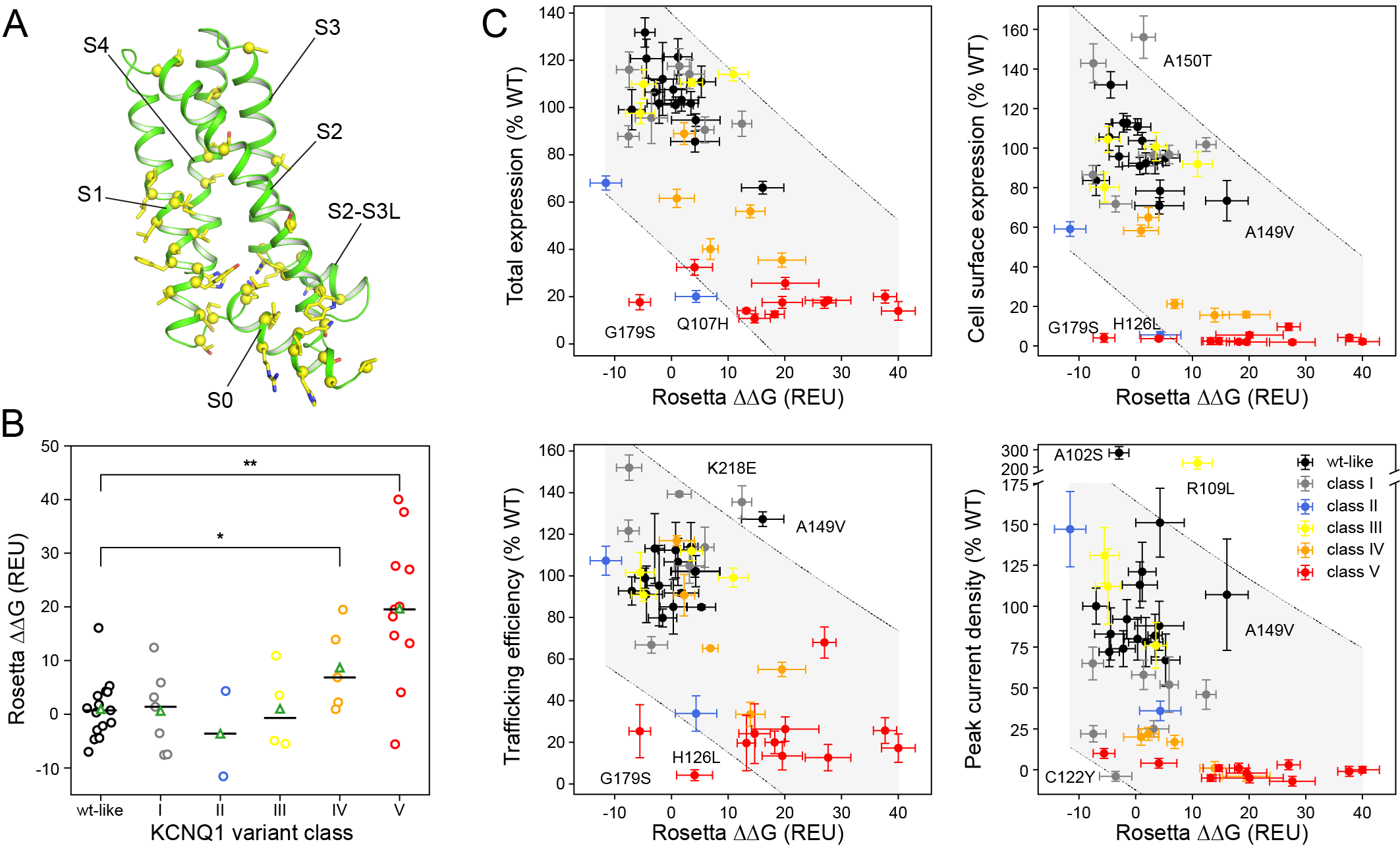

Supplement: S8 Fig — (A) Location of mutation sites in the KCNQ1 VSD. Backbone sites are mapped by yellow spheres and the native amino acid residue is indicated by yellow sticks. (B) Distribution of Rosetta ΔΔG values for the six functionally distinct classes of KCNQ1 VSD variants (i.e. wt-like and classes I to V for non-functional variants) calculated with the RC homology model. The median and average value are drawn as black horizontal line and green triangle, respectively. The median of class IV and V is compared to wt-like variants using a Kruskal-Wallis H-test (* p < 0.05, ** p < 0.01, *** p < 0.001, nwt-like = 15, nIV = 5, nV = 11). ΔΔG values for mutations to proline are off-scale (ΔΔGL114P = 77.1 ± 4.0 REU, ΔΔGL131P = 61.5 ± 2.5 REU, ΔΔGL134P = 63.7 ± 2.6 REU, ΔΔGR195P = 52.7 ± 1.1 REU, ΔΔGQ234P = 82.1 ± 3.8 REU, ΔΔGL236P = 60.3 ± 4.8 REU) due to incompatible backbone torsions in the starting model yielding bad backbone and proline ring geometries and were not used in the analysis. (C) Correlation plots of total expression level, cell surface expression, trafficking efficiency and channel peak current density versus calculated Rosetta ΔΔG values (mean ± S.E.M.). KCNQ1 variant classes are indicated with different colors. Variants that fall outside or are close to the boundary of the 95% confidence interval for a linear regression model (gray shaded area) are labeled and their structural models are shown in S8 Fig. The experimental data are from reference [19] and are listed together with the computed ΔΔG values for the RC and AO models in S2 Table. (TIFF) [file pone.0220415.s010.tiff]

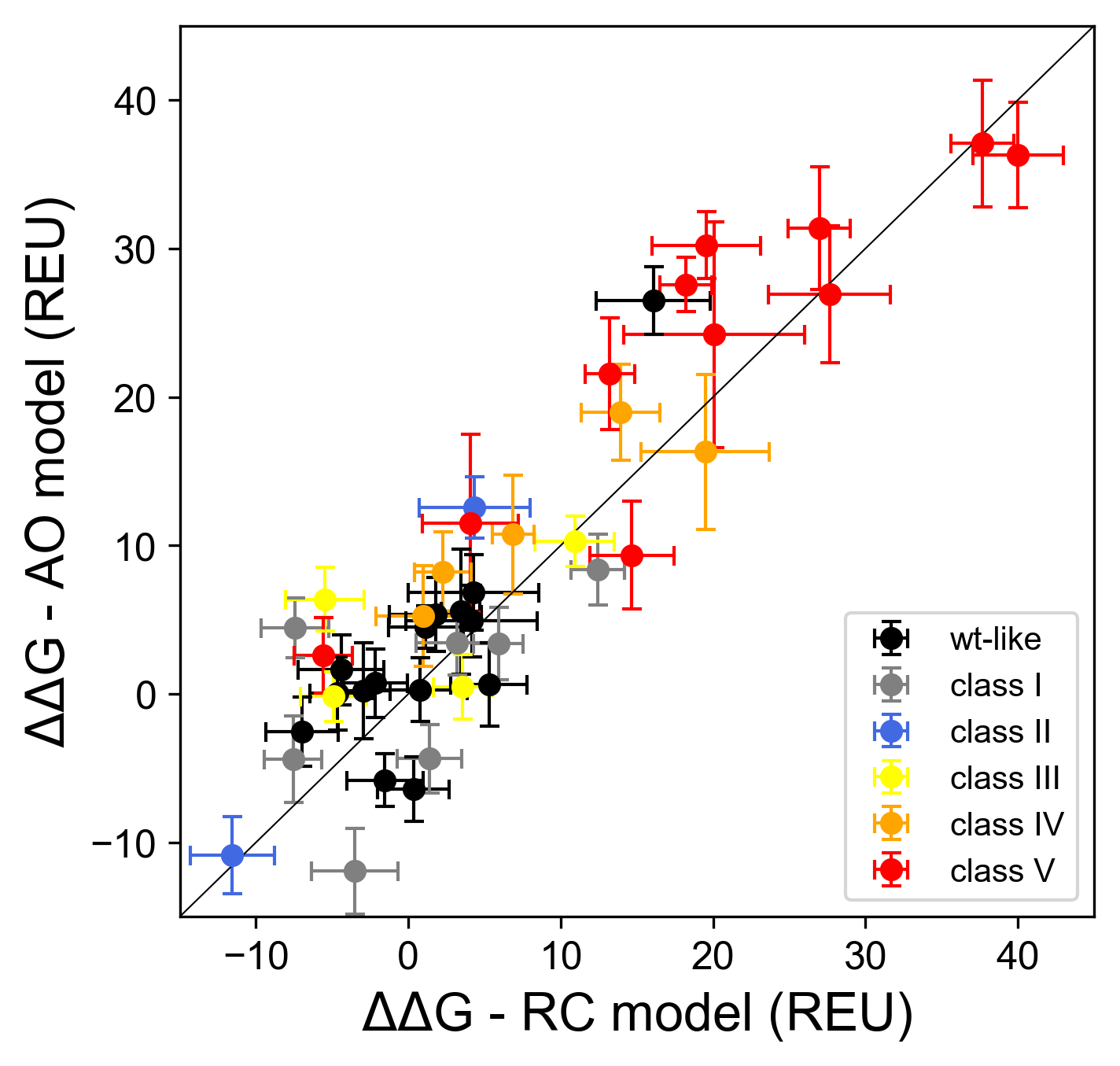

Supplement: S9 Fig — (TIFF) [file pone.0220415.s011.tiff]

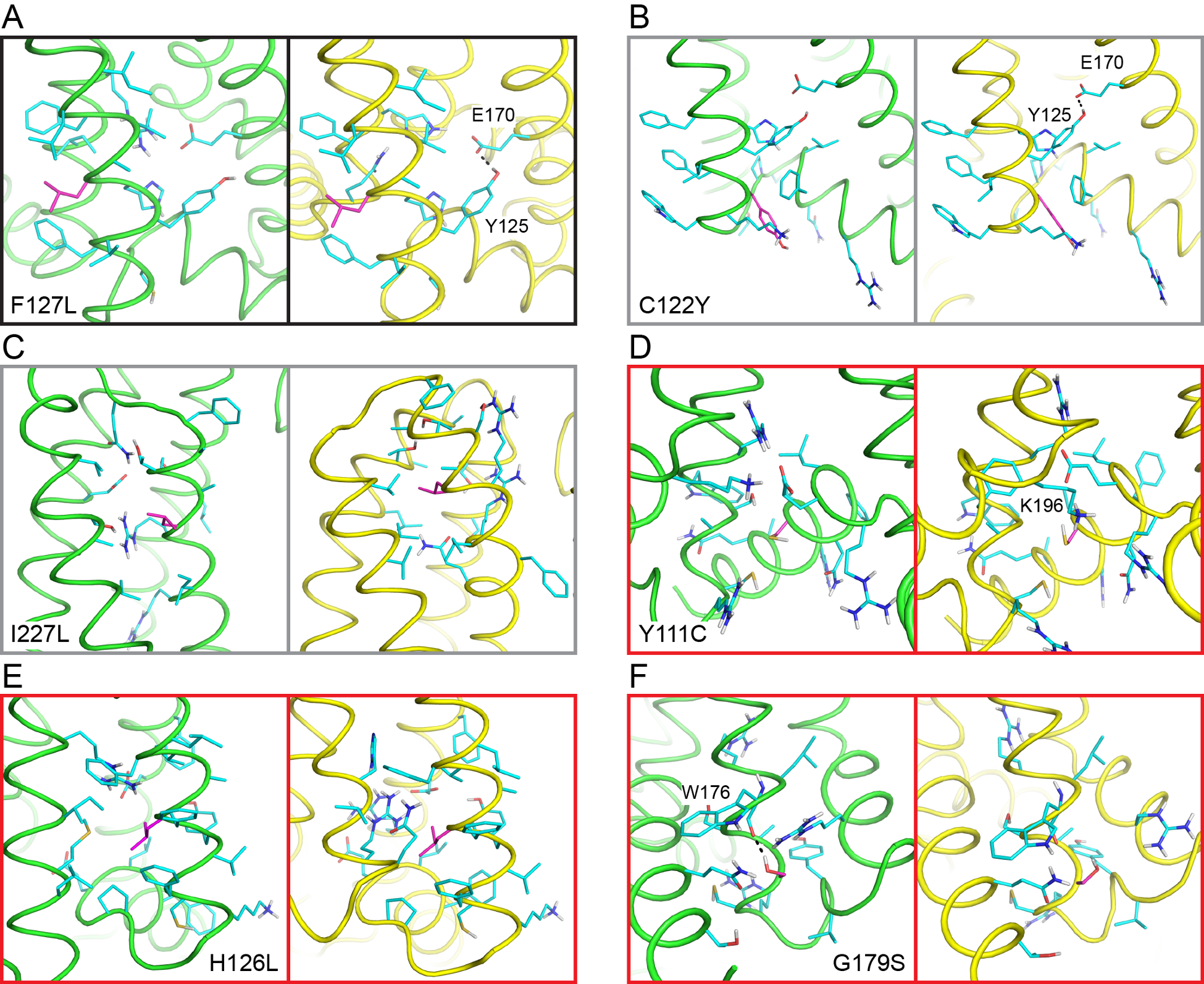

Supplement: S10 Fig — Snapshots of mutants for which varying ΔΔG predictions were obtained with the RC and AO model, respectively, are displayed. For each mutant, the backbone is drawn as cartoon (RC model: green, AO model: yellow) and the side-chains of amino acids within a 5 Å radius of the mutation site are drawn as cyan sticks. The substituted amino acid residue is colored magenta. (A) F127L: wt-like variant. The AO model (right) predicted a negative ΔΔG because of a stabilizing hydrogen bond between Y125 and E170 in the neighborhood of the mutation site which was not formed in the RC model (left). (B) C122Y: class I variant. The AO model (right) yielded a more negative ΔΔG because of a favorable attractive score (fa_atr) and a hydrogen bond between Y125 and E170 which was absent in the RC model (left). (C) I227L: class I variant. A higher ΔΔG was obtained with the AO model (right) because of unfavorable residue rotamer (fa_dun) and solvation (fa_mbsolv) scores. The mutation site is located in the S4 helix and has a different residue neighborhood and membrane insertion depth in the RC and AO state, respectively, which contributes to the observed ΔΔG differences. (D) Y111C: class V variant. A higher ΔΔG value was predicted with the AO model (right) because of more unfavorable attractive (fa_atr) and hydrogen bond (hbond_sc) scores. The increased hbond_sc score can be explained by the mutation-induced loss of a hydrogen bond with K196 which is present in wildtype AO model but absent in the RC model. (E) H126L: class V variant. The more positive ΔΔG obtained with the AO model (right) can be explained by higher residue packing density around the mutation site which led to increased attractive (fa_atr) and repulsive (fa_rep) scores upon mutation. (F) G179S: class V variant. The RC model (left) had a negative ΔΔG because of a side-chain-backbone hydrogen bond between S179 and W176 which was however absent in the AO model. (TIFF) [file pone.0220415.s012.tiff]

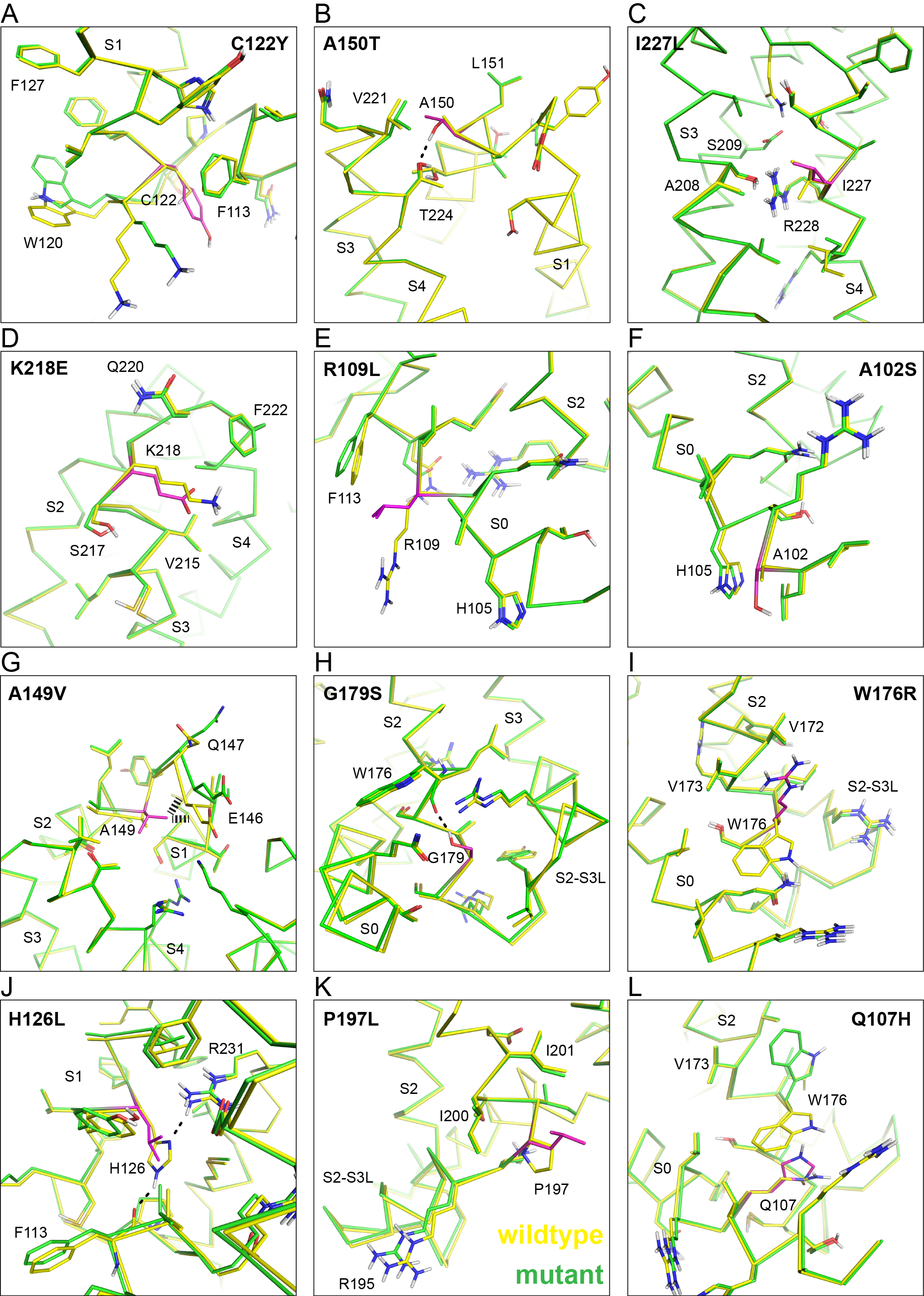

Supplement: S11 Fig — For each variant, the protein backbone is drawn as ribbon and the side-chains of amino acids within a 5 Å radius of the mutation site are drawn as sticks. The substituted amino acid residue is colored magenta. (A) C122Y: has slightly lower expression and trafficking efficiency than wildtype but the channel is not conductive. This is not reflected by the negative ΔΔG (-3.5 REU for RC model and -11.9 REU for AO model) which would indicate a stabilizing effect. (B) A150T: has increased cell surface expression and trafficking levels but a current density that is only half of that of wildtype although the protein is predicted to be as stable or more stabilized than wildtype (ΔΔG of 1.4 REU for RC model and -4.4 REU for AO model). Stabilization in the AO model arises from a sidechain-sidechain hydrogen bond with T224 in S4. (C) I227: has a negative or moderately increased ΔΔG value (-7.5 REU for RC model and 4.4 REU for AO model) and increased cell surface expression and trafficking which contrasts is very low current density. (D) K218E: has an increased positive ΔΔG value (12.8 REU and 8.4 REU for RC and AO model, respectively) (i.e. is predicted to be destabilized) and a current density that is half of that of wildtype but normal cell surface expression and increased trafficking. (E) R109L: was predicted to be destabilized (i.e. positive ΔΔG of 10.9 REU and 10.3 REU for RC and AO model, respectively), but has normal expression levels and a current density twofold higher than wildtype. (F) A102S: has negative or neutral stability change (ΔΔG of -2.9 REU and 0.2 REU for the RC and AO model, respectively) but a current density almost three times as high as wildtype. (G) A149V: belongs to wt-like class with normal or slightly reduced expression levels, but was predicted a high positive ΔΔG (16.1 REU and 26.5 REU for RC and AO model, respectively) which can be explained by steric repulsion between the Val side-chain and the nearby protein backbone in the mutant model (dashed [file pone.0220415.s013.tiff]

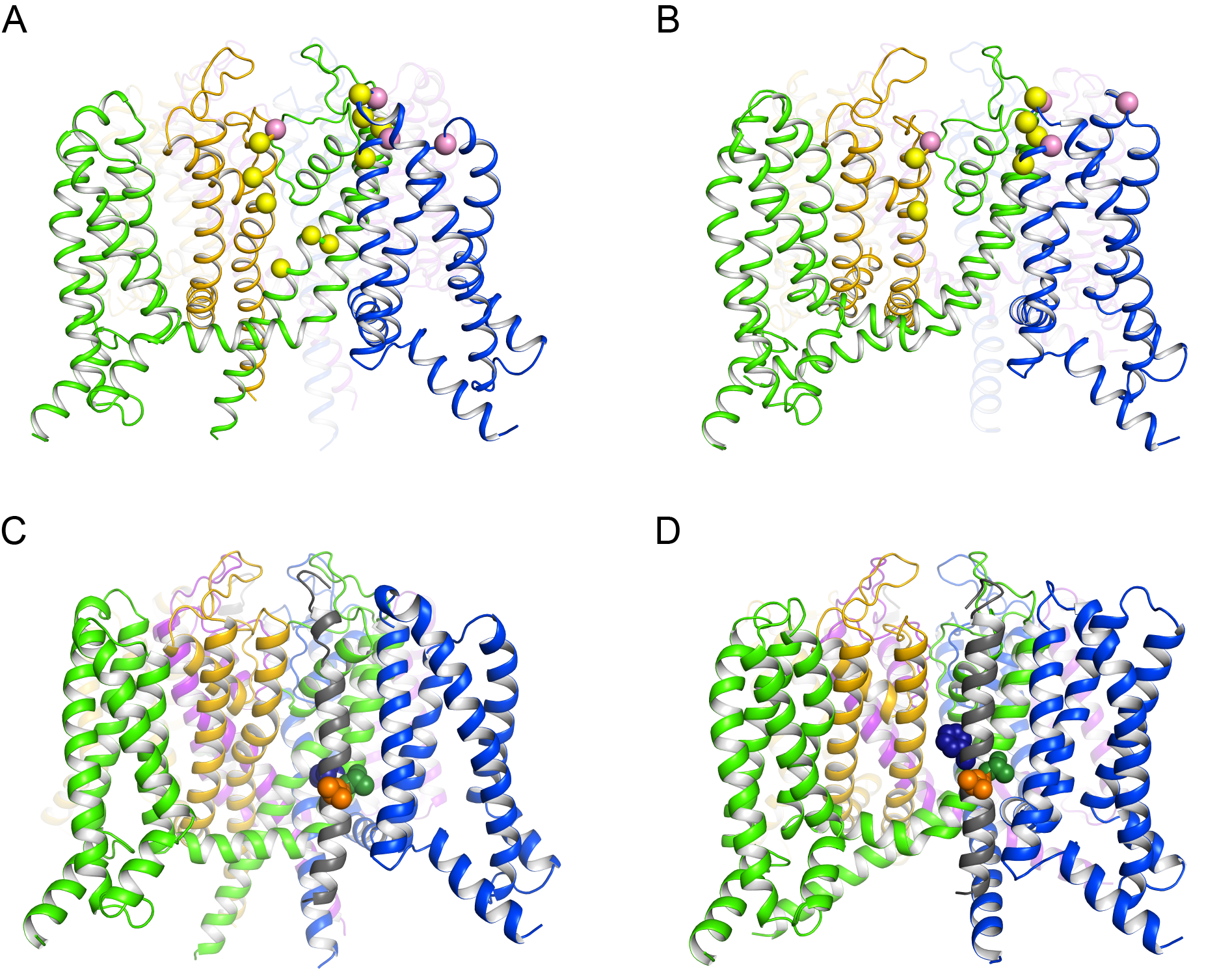

Supplement: S12 Fig — Experimental contact sites (from references [22, 23, 25, 84–87]) are mapped onto the KCNQ1 RC (A) and AO (B) models, respectively. Chain A (green), the VSD of chain B (blue) and the PD of chain D (orange) are shown in the front. The subunits in the back are drawn transparent for clarity. KCNE1 contact sites are represented as spheres. KCNQ1 positions with restraints specific for the RC or AO state, respectively, are illustrated as yellow spheres. Restraints for which the channel state was unclear are indicated as pink spheres. Panels in (C) and (D) show docking models of KCNE1 (gray) bound to KCNQ1 in the RC and AO state, respectively. The side-chains of residues Phe57, Thr58 and Leu59 are depicted as spheres and colored blue, green and orange, respectively. (TIFF) [file pone.0220415.s014.tiff]
