# Supplementary material for: Upgraded molecular models of the human KCNQ1 potassium channel
Source: PLoS One. 2019 Sep 13;14(9):e0220415. doi: 10.1371/journal.pone.0220415 (PMC6743773; doi:10.1371/journal.pone.0220415)
Supplement: S1 Input Files and Scripts — In addition, it contains a list of the used software resources and detailed method descriptions. (TAR.GZ) [file pone.0220415.s023.tar.gz › S1_Input_Files_and_Scripts/S1_software_resources_and_method_details.pdf]

A. Used Software Resources

Software + Version	Reference	Source
Rosetta3.8	A. Leaver-Fay et al. 2011, Methods Enzymol. 487:545-574	https://www.rosettacommons.org/software/license-and-download
Amber16+Ambertools17	D. A. Case et al. AMBER2016 (San Francisco: University of California, 2016)	http://ambermd.org/GetAmber.php
Gaussian09	M. J. Frisch et al. Gaussian 09 (Gaussian, Inc., Wallingford CT, 2009)	http://gaussian.com/products/
CHARMM-GUI webserver	S. Jo, T. Kim, V. G. Iyer, W. Im 2008, J. Comput. Chem. 29:1859-1865	http://www.charmm-gui.org
MolProbity webserver	I. W. Davis et al. 2007, Nucleic Acids Res. 35:W375-383	http://molprobity.biochem.duke.edu
HOLE	O. S. Smart et al. 1996, J.Mol.Graph. 14(6):354-360	http://www.holeprogram.org

B. Detailed Methods and Descriptions

1. Rosetta comparative modeling

Rosetta comparative modeling was carried out with the RosettaScripts application by running the following command and using the XML script (*rosettacm.xml*) and options file (*rosettacm.options*) listed below (Note, variables `$seed` and `$task` in the command below were initialized from the SLURM job ID and task array ID when running the calculation on a cluster).

```
~/rosetta/main/source/bin/rosetta_scripts.linuxgccrelease -database ~/rosetta/main/database \
@rosettacm.options -run:jran $seed -out:nstruct 10 -out:prefix hybridize_q1_open_"$seed"_"$task"_ \
-out:file:silent hybridize_q1_open_"$task".out > hybridize_q1_open_"$task".log &
```

Models were additionally relaxed with the Rosetta FastRelax application using the flags in *relax.options* (see below).

```
~/rosetta/main/source/bin/relax.linuxgccrelease -database ~/rosetta/main/database \
@ relax.options -in:file:silent hybridize_q1_open_"$task".out -run:jran $seed -out:nstruct 2 \
-out:file:silent relax_hybridize_q1_open_"$task".out > relax_hybridize_q1_open_"$task".log &
```

```
<ROSETTASCRIPTS>
  <TASKOPERATIONS>
  </TASKOPERATIONS>
  <SCOREFXNS>
    <ScoreFunction name="stage1" weights="stage1_membrane.wts" symmetric="1">
      <Reweight scoretype="atom_pair_constraint" weight="1"/>
    </ScoreFunction>
    <ScoreFunction name="stage2" weights="stage2_membrane.wts" symmetric="1">
      <Reweight scoretype="atom_pair_constraint" weight="0.5"/>
    </ScoreFunction>
    <ScoreFunction name="fullatom" weights="stage3_rlx_membrane.wts" symmetric="1">
      <Reweight scoretype="atom_pair_constraint" weight="0.5"/>
    </ScoreFunction>
  </SCOREFXNS>
  <FILTERS>
  </FILTERS>
```

```
  <MOVERS>
    <Hybridize name="hybridize" stage1_scorefxn="stage1" stage2_scorefxn="stage2"
       fa_scorefxn="fullatom" batch="1" stage1_increase_cycles="1.0"
       stage2_increase_cycles="1.0" linmin_only="1" realign_domains="0">
      <Fragments 3mers="Q1_open_frags.200.3mers" 9mers="Q1_open_frags.200.9mers"/>
      <Template pdb="human_on_5vms.pdb" cst_file="AUTO" weight="1.000" symmdef="Q1_open.symm"/>
      <Template pdb="human_on_2r9r.pdb" cst_file="AUTO" weight="1.000" symmdef="Q1_open.symm"/>
      Use fragments only for sampling of S3/S4 region
      <DetailedControls start_res="1" stop_res="95" sample_template="1" sample_abinitio="0"/>
      <DetailedControls start_res="96" stop_res="150" sample_template="1" sample_abinitio="1"/>
      <DetailedControls start_res="151" stop_res="270" sample_template="1" sample_abinitio="0"/>
    </Hybridize>
  </MOVERS>
```

Code 1: Rosetta XML script for comparative modeling (*rosettacm.xml*)

```
# I/O
-in:file:fasta Q1_open.fasta
-in:file:psipred_ss2 Q1_open.psipred_ss2
-parser:protocol rosettacm.xml
-out:file:silent_struct_type binary
-in:detect_disulf true
# relax options
-relax:minimize_bond_angles
-relax:minimize_bond_lengths
-relax:jump_move true
-default_max_cycles 200
-relax:min_type lbfgs_armijo_nonmonotone
-relax:jump_move true
-score:weights stage3_rlx_membrane.wts
-use_bicubic_interpolation
-hybridize:stage1_probability 1.0
-sog_upper_bound 15
# membrane options
-membrane
-in:file:spanfile Q1_open_A.span
-membrane:no_interpolate_Mpair
-membrane:Menv_penalties
-fixed_membrane true
-membrane_center 0.0 0.0 0.0
-membrane_normal 0.0 0.0 1.0
-rg_reweight .1
# symmetry options (already set in rosettacm.xml)
#-symmetry:symmetry_definition Q1_open.symm
#-symmetry:initialize_rigid_body_dofs
-mute core.io.pdb.file_data core.conformation.Conformation core.scoring.MembranePotential \
core.scoring.MembraneTopology core.scoring.CartesianBondedEnergy
```

Code 2: Rosetta options file for comparative modeling (*rosettacm.options*)

```
# I/O options
-in:file:silent_struct_type binary
-out:file:silent_struct_type binary

# specific relax options
-relax:dualspace
-relax:minimize_bond_angles
-relax:jump_move true
-relax:constrain_relax_to_start_coords true
-set_weights cart_bonded .5 pro_close 0
-default_max_cycles 200
-flip_HNQ
-no_optH false

# symmetry options
-symmetry
-symmetry_definition Q1_open.symm

# membrane options
-in:file:spanfile Q1_open_A.span
-membrane:no_interpolate_Mpair
-membrane:Menv_penalties
-fixed_membrane true
-membrane_center 0.0 0.0 0.0
-membrane_normal 0.0 0.0 1.0

-score:weights membrane_highres_Menv_smooth.wts
```

Code 3: Rosetta options file for FastRelax (*relax.options*)

2. Rosetta energy calculations of mutation-induced stability changes with FlexddG

Rosetta energy calculations with the FlexddG protocol were carried out with the RosettaScripts application. Separate energy calculations were performed for the wild type (wt) and mutant protein (mut) by using two different Rosetta XML scripts (*flexddG.wt.xml* and *flexddG.mut.xml*) and the ddG was calculated as the energy difference as explained in Materials & Methods. The Rosetta XML scripts for the wild type and mutant calculation and the options file are shown below.

```
~/rosetta/main/source/bin/rosetta_scripts.linuxgccrelease -database ~/rosetta/main/database/ \
-parser:protocol flexddG.mut.xml @ flexddG.options -in:file:s Q1_open_1.pdb \
-packing:resfile C122Y.resfile -out:file:scorefile Q1_open_C122Y.fsc \
-out:file:silent Q1_open_C122Y.out -out:nstruct 50 > Q1_open_C122Y.log &
```

```
~/rosetta/main/source/bin/rosetta_scripts.linuxgccrelease -database ~/rosetta/main/database/ \
-parser:protocol flexddG.wt.xml @ flexddG.options -in:file:s Q1_open_1.pdb \
-packing:resfile C122Y.resfile -out:file:scorefile Q1_open_C122wt.fsc \
-out:file:silent Q1_open_C122wt.out -out:nstruct 50 > Q1_open_C122wt.log &
```

The Resfile (e.g. *C122Y.resfile*) had the following format:

```
NATAA
start

122 A PIKAA Y
```

For protein minimization with the soft repulsive score function the fa_rep term was scaled down by applying the following patch files (see `ScoreFunction` section in the Rosetta XML script)

min_soft1.wts_patch

```
fa_rep *= 0.1
```

min_soft2.wts_patch

```
fa_rep *= 0.333
```

```
<ROSETTASCRIPTS>
  <SCOREFXNS>
    <ScoreFunction name="sfxn_min_soft1" weights="%%min_sfxn%%" symmetric="1"
     patch="%%min_soft1_patch%%">
      <Reweight scoretype="atom_pair_constraint" weight="1.0"/>
      <Set fa_max_dis="9.0"/>
    </ScoreFunction>
    <ScoreFunction name="sfxn_min_soft2" weights="%%min_sfxn%%" symmetric="1"
     patch="%%min_soft2_patch%%">
      <Reweight scoretype="atom_pair_constraint" weight="1.0"/>
      <Set fa_max_dis="9.0"/>
    </ScoreFunction>
    <ScoreFunction name="sfxn_min_hard" weights="%%min_sfxn%%" symmetric="1">
      <Reweight scoretype="atom_pair_constraint" weight="1.0"/>
      <Set fa_max_dis="9.0"/>
    </ScoreFunction>
    <ScoreFunction name="sfxn_pack_soft" weights="%%soft_pack_sfxn%%" symmetric="1">
      <Reweight scoretype="atom_pair_constraint" weight="1.0"/>
    </ScoreFunction>
    <ScoreFunction name="sfxn_mem" weights="%%output_sfxn%%" symmetric="1">
    </ScoreFunction>
  </SCOREFXNS>

  <TASKOPERATIONS>
    <ReadResfile name="rrf"/>
  </TASKOPERATIONS>

  <RESIDUE_SELECTORS>
    <Task name="mutsite" fixed="0" packable="0" designable="1" task_operations="rrf"/>
    <Neighborhood name="mutsite_nbrs" selector="mutsite" distance="8.0"/>
    <PrimarySequenceNeighborhood name="mutsite_nbrs_adjacent" selector="mutsite_nbrs"
     lower="1" upper="1"/>
    <StoredResidueSubset name="restore_nbr_shell" subset_name="nbr_shell"/>
    <Not name="not_nbr_shell" selector="restore_nbr_shell"/>
  </RESIDUE_SELECTORS>

  <TASKOPERATIONS>
    <RestrictToRepacking name="rtr"/>
    <InitializeFromCommandline name="ifcl"/>
    <ExtraRotamersGeneric name="ex1ex2" ex1="1" ex2="1" extrachi_cutoff="0"/>
    <OperateOnResidueSubset name="repack" selector="restore_nbr_shell">
      <RestrictToRepackingRLT/>
    </OperateOnResidueSubset>
    <OperateOnResidueSubset name="norepack" selector="not_nbr_shell">
      <PreventRepackingRLT/>
    </OperateOnResidueSubset>
  </TASKOPERATIONS>

  <FILTERS>
    <ScoreType name="score_filter" scorefxn="sfxn_min_hard" score_type="total_score"
     threshold="99990.9"/>
  </FILTERS>

  <MOVERS>
    INITIAL SETUP
```

```
    <StoreResidueSubset name="store_nbr_shell" subset_name="nbr_shell"
      residue_selector="mutsite_nbrs_adjacent"/>
    <SetupForSymmetry name="setup_symmetry" definition="%%symdef_file%%"/>
    <ScoreMover name="apply_score" scorefxn="sfxn_mem" verbose="0"/>

    CONSTRAINTS
    <AddConstraintsToCurrentConformationMover name="addcst" use_distance_cst="1" coord_dev="0.5"
      min_seq_sep="0" max_distance="9" CA_only="1" bound_width="0.0" cst_weight="1.0"/>
    <ClearConstraintsMover name="clearcst"/>

    BACKRUB
    <BackrubProtocol name="backrub" mc_kt="1.2" ntrials="15000"
      pivot_residue_selector="restore_nbr_shell" task_operations="ifcl,ex1ex2,rtr"
      recover_low="0" trajectory="0" trajectory_stride="500"/>

    PACKING AND MINIMIZATION
    <SymPackRotamersMover name="mutate" scorefxn="sfxn_pack_soft"
      task_operations="ifcl,ex1ex2,rrf,norepack"/>
    <SymPackRotamersMover name="repack" scorefxn="sfxn_pack_soft"
      task_operations="ifcl,ex1ex2,repack,norepack"/>
    <SymMinMover name="min_soft1" scorefxn="sfxn_min_soft1" bb="1" chi="1" jump="ALL"
      cartesian="0" type="lbfgs_armijo_nonmonotone" tolerance="0.000001" max_iter="5000"/>
    <SymMinMover name="min_soft2" scorefxn="sfxn_min_soft2" bb="1" chi="1" jump="ALL"
      cartesian="0" type="lbfgs_armijo_nonmonotone" tolerance="0.000001" max_iter="5000"/>
    <SymMinMover name="min_hard" scorefxn="sfxn_min_hard" bb="1" chi="1" jump="ALL"
      cartesian="0" type="lbfgs_armijo_nonmonotone" tolerance="0.000001" max_iter="5000"/>
    <ParsedProtocol name="min_seq" mode="sequence">
    <Add mover="min_soft1"/>
      <Add mover="min_soft2"/>
      <Add mover="min_hard"/>
    </ParsedProtocol>
    <IteratedConvergence name="minimize_until_converged" mover="min_seq" filter="score_filter"
      delta="1.0" cycles="1" maxcycles="50"/>
  </MOVERS>

  <APPLY_TO_POSE>
  </APPLY_TO_POSE>
  <PROTOCOLS>
    Setup symmetric pose and do initial minimize
    <Add mover="setup_symmetry"/>
    <Add mover="addcst"/>
    <Add mover="apply_score"/>
    <Add mover="store_nbr_shell"/>
    <Add mover="min_hard"/>
    <Add mover="clearcst"/>
    Backrub, pack and minimize
    <Add mover="backrub"/>
    Add mover="mutate"/>
    <Add mover="repack"/>
    <Add mover="addcst"/>
    <Add mover="minimize_until_converged"/>
    Remove constraints and calculate binding energy
    <Add mover="clearcst"/>
  </PROTOCOLS>
  <OUTPUT scorefxn="sfxn_mem"/>
</ROSETTASCRIPTS>
```

Code 4: Rosetta XML script for the mutant energy calculation (*flexddG.mut.xml*)

```
<ROSETTASCRIPTS>
  <SCOREFXNS>
    <ScoreFunction name="sfxn_min_soft1" weights="%%min_sfxn%%" symmetric="1"
       patch="%%min_soft1_patch%%">
      <Reweight scoretype="atom_pair_constraint" weight="1.0"/>
      <Set fa_max_dis="9.0"/>
    </ScoreFunction>
    <ScoreFunction name="sfxn_min_soft2" weights="%%min_sfxn%%" symmetric="1"
       patch="%%min_soft2_patch%%">
      <Reweight scoretype="atom_pair_constraint" weight="1.0"/>
      <Set fa_max_dis="9.0"/>
    </ScoreFunction>
    <ScoreFunction name="sfxn_min_hard" weights="%%min_sfxn%%" symmetric="1">
      <Reweight scoretype="atom_pair_constraint" weight="1.0"/>
      <Set fa_max_dis="9.0"/>
    </ScoreFunction>
    <ScoreFunction name="sfxn_pack_soft" weights="%%soft_pack_sfxn%%" symmetric="1">
      <Reweight scoretype="atom_pair_constraint" weight="1.0"/>
    </ScoreFunction>
    <ScoreFunction name="sfxn_mem" weights="%%output_sfxn%%" symmetric="1">
    </ScoreFunction>
  </SCOREFXNS>

  <TASKOPERATIONS>
    <ReadResfile name="rrf"/>
  </TASKOPERATIONS>

  <RESIDUE_SELECTORS>
    <Task name="mutsite" fixed="0" packable="0" designable="1" task_operations="rrf"/>
    <Neighborhood name="mutsite_nbrs" selector="mutsite" distance="8.0"/>
    <PrimarySequenceNeighborhood name="mutsite_nbrs_adjacent" selector="mutsite_nbrs"
       lower="1" upper="1"/>
    <StoredResidueSubset name="restore_nbr_shell" subset_name="nbr_shell"/>
    <Not name="not_nbr_shell" selector="restore_nbr_shell"/>
  </RESIDUE_SELECTORS>

  <TASKOPERATIONS>
    <RestrictToRepacking name="rtr"/>
    <InitializeFromCommandline name="ifcl"/>
    <ExtraRotamersGeneric name="ex1ex2" ex1="1" ex2="1" extrachi_cutoff="0"/>
    <OperateOnResidueSubset name="repack" selector="restore_nbr_shell">
      <RestrictToRepackingRLT/>
    </OperateOnResidueSubset>
    <OperateOnResidueSubset name="norepack" selector="not_nbr_shell">
      <PreventRepackingRLT/>
    </OperateOnResidueSubset>
  </TASKOPERATIONS>

  <FILTERS>
    <ScoreType name="score_filter" scorefxn="sfxn_min_hard" score_type="total_score"
       threshold="99990.9"/>
  </FILTERS>

  <MOVERS>
    INITIAL SETUP
    <StoreResidueSubset name="store_nbr_shell" subset_name="nbr_shell"
       residue_selector="mutsite_nbrs_adjacent"/>
    <SetupForSymmetry name="setup_symmetry" definition="%%symdef_file%%"/>
    <ScoreMover name="apply_score" scorefxn="sfxn_mem" verbose="0"/>

    CONSTRAINTS
    <AddConstraintsToCurrentConformationMover name="addcst" use_distance_cst="1"
       coord_dev="0.5" min_seq_sep="0" max_distance="9" CA_only="1" bound_width="0.0"
       cst_weight="0.0"/>
    <ClearConstraintsMover name="clearcst"/>
```

```
    BACKRUB
    <BackrubProtocol name="backrub" mc_kt="1.2" ntrials="15000"
       pivot_residue_selector="restore_nbr_shell" task_operations="ifcl,ex1ex2,rtr"
       recover_low="0" trajectory="0" trajectory_stride="500"/>

    PACKING AND MINIMIZATION
    <SymPackRotamersMover name="mutate" scorefxn="sfxn_pack_soft"
       task_operations="ifcl,ex1ex2,rrf,norepack"/>
    <SymPackRotamersMover name="repack" scorefxn="sfxn_pack_soft"
       task_operations="ifcl,ex1ex2,repack,norepack"/>
    <SymMinMover name="min_soft1" scorefxn="sfxn_min_soft1" bb="1" chi="1" jump="ALL"
       cartesian="0" type="lbfgs_armijo_nonmonotone" tolerance="0.000001"
       max_iter="5000"/>
    <SymMinMover name="min_soft2" scorefxn="sfxn_min_soft2" bb="1" chi="1" jump="ALL"
       cartesian="0" type="lbfgs_armijo_nonmonotone" tolerance="0.000001"
       max_iter="5000"/>
    <SymMinMover name="min_hard" scorefxn="sfxn_min_hard" bb="1" chi="1" jump="ALL"
       cartesian="0" type="lbfgs_armijo_nonmonotone" tolerance="0.000001"
       max_iter="5000"/>
    <ParsedProtocol name="min_seq" mode="sequence">
      <Add mover="min_soft1"/>
      <Add mover="min_soft2"/>
      <Add mover="min_hard"/>
    </ParsedProtocol>
    <IteratedConvergence name="minimize_until_converged" mover="min_seq"
       filter="score_filter" delta="1.0" cycles="1" maxcycles="50"/>

  </MOVERS>
  <APPLY_TO_POSE>
  </APPLY_TO_POSE>
  <PROTOCOLS>
    Setup symmetric pose and do initial minimize
    <Add mover="setup_symmetry"/>
    <Add mover="addcst"/>
    <Add mover="apply_score"/>
    <Add mover="store_nbr_shell"/>
    <Add mover="min_hard"/>
    <Add mover="clearcst"/>
    Backrub, pack and minimize
    <Add mover="backrub"/>
    <Add mover="mutate"/>
    Add mover="repack"/>
    <Add mover="addcst"/>
    <Add mover="minimize_until_converged"/>
    Remove constraints and calculate binding energy
    <Add mover="clearcst"/>
  </PROTOCOLS>
  <OUTPUT scorefxn="sfxn_mem"/>
</ROSETTASCRIPTS>
```

Code 5: Rosetta XML script for the wild type energy calculation (*flexddG.wt.xml*)

```
-out:file:silent_struct_type binary
# membrane options
-in:file:spanfile Q1_open_A.span
-membrane:no_interpolate_Mpair
-membrane:Menv_penalties
-fixed_membrane true
-membrane_center 0.0 0.0 0.0
-membrane_normal 0.0 0.0 1.0
# symmetry options
-symmetry:symmetry_definition Q1_open.symm
-symmetry:initialize_rigid_body_dofs
-parser:script_vars symdef_file=Q1_open.symm \
min_sfxn=membrane_highres_Menv_smooth.wts soft_pack_sfxn=soft_rep_design.wts \
output_sfxn=membrane_highres_Menv_smooth.wts min_soft1_patch=min_soft1.wts_patch \
min_soft2_patch=min_soft2.wts_patch

-corrections:restore_talaris_behavior
-score:weights membrane_highres_Menv_smooth.wts
-out:mute core.scoring.MembraneTopology
```

Code 6: Rosetta options file for energy calculation with the FlexddG protocol (*flexddG.options*)

3. Amber MD simulations

The Rosetta models were embedded in a POPC/PIP2 membrane bilayer using the membrane builder tool of the CHARMM-GUI website (http://www.charmm-gui.org/?doc=input/membrane.bilayer). The PDB file of the created protein-membrane MD system after step 5 of the membrane builder tool was converted into an Amber PDB file by following the instructions from the Amber Lipid Tutorial (http://ambermd.org/tutorials/advanced/tutorial16/index.html) and converted into Amber topology and coordinate files using Leap (see Leap input script below). The library object file (*pip2.lib*) and the force field modification file (*pip2.frcmod*) are provided with the other MD input files in the paper supplement. Minimization, heating and equilibration of the MD system, and production MD were run on GPUs on a cluster using Amber's PMEMD engine. The input scripts for every simulation step are provided as extra files (because their length exceeds several pages) in the paper supplement.

```
source leaprc.ff14SB
source leaprc.water.tip3p
source leaprc.lipid17
loadamberparams frcmod.ionsjc_tip3p
loadoff pip2.lib # library with PI4 and PI5 residue
loadamberparams pip2.frcmod # additional force field parameters for PI4 and PI4
kcnq1 = loadpdb kcnq1_rc_model_charmm_gui.pdb
set kcnq1 box { 154.184 153.915 111.806 } # Box size was measured in VMD
saveamberparm kcnq1 kcnq1_rc_amber.prmtop kcnq1_rc_amber.inpcrd
quit
EOF
```

Code 7: Leap input script for creating Amber topology and coordinate files
